# Association of the Albumin–Bilirubin score with 7-day incident delirium risk following bloodstream infection in critically ill adults: evidence from a propensity-weighted cohort

**DOI:** 10.1186/s12879-026-13228-3

**Published:** 2026-04-03

**Authors:** Ye Fang, Jiang-Tao Deng, Jiao Xu

**Affiliations:** 1 Department of Anesthesiology, The Eighth Affiliated Hospital of Sun Yat-sen University, Shenzhen, Guangdong 518000 China; 2 Department of Critical Care Medicine, The University of Hong Kong-Shenzhen Hospital, Shenzhen, Guangdong 518000 China; 3 Department of Pain Medicine, Xiangyang Central Hospital, Affiliated Hospital of Hubei University of Arts and Science, Xiangyang, Hubei 441000 China

**Keywords:** Albumin–bilirubin (ALBI) score, Delirium, Bloodstream infection, Risk stratification, Critical care database

## Abstract

**Background:**

Delirium is a frequent complication in patients with bloodstream infection (BSI) and is associated with poor outcomes. The albumin–bilirubin (ALBI) score, an evidence-based index of hepatic function and systemic inflammation, has shown prognostic value in sepsis. However, its association with the risk of incident delirium in patients with BSI remains unclear. We aimed to evaluate the utility of the ALBI score in stratifying the risk of incident delirium in this population.

**Methods:**

We conducted a retrospective cohort study using the Medical Information Mart for Intensive Care IV database (MIMIC-IV, v3.1) involving adult patients admitted to the intensive care unit (ICU) with a first episode of BSI. The primary exposure was the baseline ALBI score, and the primary outcome was incident delirium within 7 days. To rigorously control for confounding, we adopted a doubly robust estimation strategy. Inverse probability of treatment weighting (IPTW) based on covariate-balancing propensity scores (CBPS) was applied to maximize the balance of baseline characteristics between groups. Subsequently, multivariable Fine-Gray proportional subdistribution hazard models were employed to estimate subdistribution hazard ratios (sHRs), accounting for death as a competing risk and adjusting for residual confounding using covariates identified via a hybrid feature selection strategy. Additionally, restricted cubic spline (RCS) analysis was performed to characterize dose–response relationships, and subgroup analyses were conducted to evaluate consistency across clinical strata.

**Results:**

A total of 651 patients were included and stratified into low- and high-ALBI groups based on a median cutoff of -1.51. The high-ALBI group had a significantly higher 7-day cumulative incidence of delirium compared to the low-ALBI group (56.4% vs. 44.3%, Gray’s test *P* = 0.004). In the fully adjusted Fine-Gray model, each 1-unit increase in the ALBI score was independently associated with an increased risk of delirium (sHR 1.80, 95% CI 1.38–2.33, *P* < 0.001). Categorical analysis confirmed that patients in the high-ALBI group had nearly double the risk (sHR 1.98, 95% CI 1.38–2.83, *P* < 0.001). RCS analysis revealed a non-linear, J-shaped dose–response relationship. Subgroup analyses demonstrated that these associations remained largely consistent across various clinical strata, with no significant interactions identified (P for interaction > 0.05).

**Conclusions:**

Higher baseline ALBI scores are independently associated with an increased risk of 7-day incident delirium in critically ill adults with BSI. Derived from routinely available laboratory tests, the ALBI score may serve as a complementary objective marker for early risk stratification identifying patients who may benefit from preventive interventions, rather than a standalone predictive tool.

**Clinical trial number:**

Not applicable.

**Supplementary Information:**

The online version contains supplementary material available at 10.1186/s12879-026-13228-3.

## Introduction

Delirium is a frequent and consequential manifestation of sepsis-associated acute brain dysfunction, occurring in approximately 20–50% of critically ill patients [1–3] and at an even higher rate among those with bloodstream infection (BSI) [4, 5]. In this context, delirium is not only a transient neuropsychiatric syndrome but also a predictor of poor outcomes, including increased short-term mortality [6], prolonged intensive care unit (ICU) stay [7], higher healthcare costs [8], and long-term cognitive decline [9]. These adverse consequences highlight the urgent need for early and objective tools to stratify delirium risk, especially in populations with BSI where systemic inflammation and sepsis-associated encephalopathy are common.

Biomarker-based approaches could complement clinical screening tools such as the Confusion Assessment Method for the ICU (CAM-ICU), offering an objective, reproducible means of risk prediction. However, in current practice, validated biomarkers for delirium remain limited. The albumin–bilirubin (ALBI) score—calculated from routinely measured serum albumin and total bilirubin—was initially developed as an evidence-based index of hepatic functional reserve in hepatology [10] but has since gained broader application [11]. Recent studies suggest that higher ALBI values are associated with adverse outcomes in sepsis, including higher mortality and organ dysfunction, underscoring its potential prognostic value beyond liver disease [12]. Mechanistically, hypoalbuminemia may exacerbate systemic inflammation, oxidative stress [13, 14], and BBB disruption [15], while elevated bilirubin can cross a compromised blood–brain barrier (BBB) and exert direct neurotoxic effects [16]. These pathways overlap with the biological processes underlying sepsis-associated encephalopathy and delirium, suggesting that ALBI may serve as a biologically plausible marker for brain vulnerability in sepsis.

Despite these insights, the association between ALBI and delirium in patients with BSI has not been systematically evaluated. Given the high incidence of delirium and its profound clinical consequences, identifying a simple and widely available biomarker to stratify risk could have important implications for ICU management. We therefore hypothesized that higher baseline ALBI at the time of the index positive blood culture would be associated with an increased 7-day risk of incident delirium in a graded exposure–response pattern. To test this hypothesis, we conducted a retrospective cohort analysis using a large, high-quality critical care database and applied robust statistical adjustment and sensitivity analyses to minimize confounding and strengthen causal inference.

## Methods

### Data source

This retrospective cohort study utilized data from the Medical Information Mart for Intensive Care IV (MIMIC-IV) database (version 3.1), which contains detailed clinical information for patients admitted to Beth Israel Deaconess Medical Center from 2008 to 2019. All personal identifiers were removed to ensure confidentiality. The study was exempt from informed consent and ethical approval requirements. The investigator (Deng) completed the required data use training (Certificate No. 61424658) and obtained authorized access to the database.

### Study population

BSI and Delirium Assessment Bloodstream infection was defined as the presence of pathogenic bacteria isolated from blood culture. Microbiological data were extracted from the microbiologyevents table. To ensure diagnostic accuracy and exclude potential contamination (e.g., coagulase-negative Staphylococci, Corynebacterium), we adhered to the Centers for Disease Control and Prevention (CDC) criteria requiring at least two separate positive cultures within 48 h for common skin commensals [17]. Delirium was assessed using the CAM-ICU, which evaluates four domains: (1) acute onset or fluctuating course, (2) inattention, (3) disorganized thinking, and (4) altered level of consciousness. A diagnosis of delirium required the presence of both domains 1 and 2, together with either domain 3 or 4. Information on delirium assessment was extracted from the chartevents table in MIMIC-IV. Specifically, delirium cases were identified based on recorded values of “Positive” or “Yes” under ItemIDs 228,303 and 228,332.

**Fig. 1:**
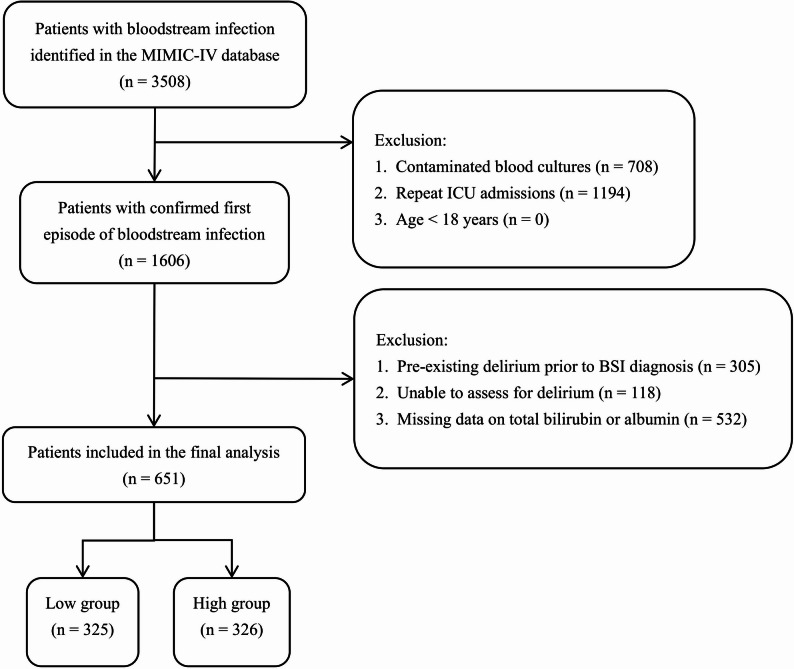
Flowchart of patient recruitment and selection. A total of 651 patients with bloodstream infection at risk of delirium were finally included from the MIMIC-IV database. The cohort was stratified into low and high ALBI score groups based on a median cutoff value of -1.51. Abbreviations: MIMIC-IV, Medical Information Mart for Intensive Care IV; ICU, intensive care unit; ALBI, albumin-bilirubin

The patient selection process is shown in Fig. 1. Of the 3,508 patients with BSI at risk of delirium initially identified from the MIMIC-IV database, 708 were excluded due to contaminated blood cultures (defined as the isolation of common skin commensals without a second positive culture within 48 h), and 1,194 were excluded due to repeat ICU admissions. No patients were excluded for age < 18 years. Consequently, 1,606 patients with a confirmed first episode of BSI were eligible for further screening. From this cohort, the following exclusions were applied: 305 patients with pre-existing delirium prior to BSI diagnosis, 118 patients who were unable to be assessed for delirium, and 532 patients with missing data on total bilirubin or albumin. To assess potential selection bias, a comparison of baseline characteristics between the patients excluded due to missing data on total bilirubin or albumin and the included study cohort is provided in Supplementary Table S1. Ultimately, 651 adult patients were included in the final analysis. To ensure balanced group sizes for statistical comparison, patients were stratified by the median ALBI score of -1.51 into the low group (*n* = 325) and the high group (*n* = 326).

### Data extraction and processing

All clinical and laboratory variables were extracted from the MIMIC-IV database. Baseline characteristics included demographics (age, gender) and major comorbidities, specifically diabetes mellitus (DM), hypertension (HTN), heart failure (HF), chronic kidney disease (CKD), and chronic obstructive pulmonary disease (COPD). Severity of illness scores, including the Sequential Organ Failure Assessment (SOFA) and Glasgow Coma Scale (GCS), were also calculated. Vital signs (body temperature, oxygen saturation) and a comprehensive panel of laboratory parameters were extracted within the first 24 h following the index positive blood culture and prior to the assessment of the primary outcome (incident delirium), ensuring the temporal sequence of exposure and outcome. Hematological variables consisted of white blood cell (WBC) count with differential, red blood cell (RBC) count, hemoglobin (Hb), hematocrit (Hct), platelet count (PLT), mean corpuscular volume (MCV), mean corpuscular hemoglobin (MCH), mean corpuscular hemoglobin concentration (MCHC), and red cell distribution width (RDW/RDW-SD). Biochemical and electrolyte profiles included sodium (Na), potassium (K), chloride, bicarbonate (HCO₃), urea nitrogen (BUN), creatinine (Cr), glucose, magnesium (Mg), phosphate (Phos), total and free calcium (Ca), albumin, total bilirubin, anion gap (AG), and lactate. Liver and cardiac markers comprised alanine aminotransferase (ALT), aspartate aminotransferase (AST), alkaline phosphatase (ALP), lactate dehydrogenase (LDH), creatine kinase (CK), CK-MB, and troponin T. Coagulation assessments included prothrombin time (PT), partial thromboplastin time (PTT), international normalized ratio (INR), and fibrinogen. Arterial blood gas parameters included pH, partial pressure of oxygen (PaO₂), partial pressure of carbon dioxide (PaCO₂), total CO₂, and base excess. Urinalysis parameters included specific gravity, pH, protein, glucose, ketones, bilirubin, urobilinogen, nitrite, leukocyte esterase, and microscopic findings. To rigorously account for predisposing and precipitating factors of delirium, we specifically extracted history of dementia (identified using ICD-9 and ICD-10 diagnosis codes for Alzheimer’s disease, vascular dementia, and other specified dementias) and sedative and analgesic exposure (defined as the administration of midazolam, lorazepam, propofol, fentanyl, or morphine within the 24 h preceding the first positive CAM-ICU assessment). The ALBI score was calculated using total bilirubin and albumin, according to the following formula: ALBI score = (log₁₀ [TBil, µmol/L] × 0.66) + (Albumin [g/L] × −0.085).

#### Outcome definition

The primary outcome was incident delirium within 7 days following the diagnosis of BSI. To strictly isolate incident cases, patients with a positive CAM-ICU assessment or a documented history of delirium in the 24 h prior to the diagnosis of BSI were excluded from the analysis. Death prior to the occurrence of delirium was considered a competing risk.

#### Data preprocessing

To preserve genuine pathological values consistent with the severity of sepsis, outliers were handled using clinically plausible range filtering based on domain knowledge, rather than purely statistical truncation (e.g., boxplot-based filtering), which might inadvertently exclude severe but valid physiological derangements. Continuous variables with > 20% missingness were excluded from the analysis. Remaining missing values were imputed using the MissForest algorithm, a non-parametric imputation method based on random forests. Unordered categorical variables were processed using one-hot encoding. Multicollinearity was assessed using the variance inflation factor (VIF; Table S2) and pairwise Pearson correlations (Figure S1); variables with a VIF > 5 or a correlation coefficient |r| > 0.70 were excluded to ensure model stability.

### Covariate selection

Covariates for the multivariable Fine-Gray proportional subdistribution hazard models were determined using a hybrid approach that combined rigorous statistical feature selection with clinical expertise. First, to identify robust data-driven predictors, we implemented a dual-feature selection strategy utilizing two distinct algorithms: (1) LASSO regression to select a parsimonious set of features, and (2) the Boruta algorithm to identify all relevant features. The intersection of both algorithms identified the following variables: COPD and specific laboratory indices (ALBI score, MCHC, MCV, RDW, phosphate, anion gap, urea nitrogen, bicarbonate, and sodium).

Second, to ensure clinical interpretability and adjust for established confounders, demographics (age and gender), major comorbidities (diabetes mellitus, hypertension, heart failure, and chronic kidney disease), and critical risk factors for delirium (history of dementia and sedative/analgesic exposure) were included in the final models a priori based on clinical experience and previous literature, regardless of their algorithmic selection status.

### Statistical analysis

Continuous variables were tested for normality using the Shapiro–Wilk test (or Anderson-Darling test for large samples). Non-normally distributed variables were presented as medians with interquartile ranges [IQRs] and compared between groups using the Mann–Whitney U test. Categorical variables were presented as counts and percentages and compared using the chi-square test. Standardized mean differences (SMDs) were calculated to assess covariate balance before and after weighting. All analyses were performed using R software (version 4.4.1; R Foundation for Statistical Computing).

Inverse probability of treatment weighting (IPTW) based on the covariate balancing propensity score (CBPS) was applied to account for baseline differences between groups. After weighting, covariate balance was assessed using SMDs, with values < 0.1 indicating adequate balance.

Considering death as a competing risk for delirium, the cumulative incidence of delirium was estimated using the cumulative incidence function (CIF), and differences between groups were assessed with Gray’s test. IPTW-adjusted multivariable Fine-Gray proportional subdistribution hazard models were applied to evaluate the association between ALBI score and the risk of delirium. Three models were constructed: (1) unadjusted; (2) adjusted for age and gender; and (3) fully adjusted for demographic, comorbidity, clinical, and laboratory covariates selected by the hybrid feature selection strategy. Results were reported as subdistribution hazard ratios (sHRs) with 95% confidence intervals (CIs). Restricted cubic spline (RCS) analysis based on the Fine-Gray model was performed to explore potential non-linear relationships between ALBI score and delirium risk. Predefined subgroup analyses were conducted to assess the consistency of associations across clinically relevant strata, with results presented as forest plots. A two-tailed p-value < 0.05 was considered statistically significant.

## Results

### Baseline characteristics

Among 651 adults with BSI included in the final analysis, 325 were classified into the low-ALBI group and 326 into the high-ALBI group. Before weighting, patients in the high-ALBI group exhibited a markedly more severe biochemical profile, characterized by greater hepatic dysfunction and coagulopathy—indicated by higher AST (70.5 vs. 40.3 U/L), ALP (140.3 vs. 101.0 U/L), INR (1.60 vs. 1.40), and PTT (37.5 vs. 33.3 s)—along with more profound metabolic derangements, including lower HCO₃⁻ (21.2 vs. 22.8 mmol/L), lower total calcium (8.02 vs. 8.53 mg/dL), and higher lactate (2.29 vs. 1.90 mmol/L) (all *P* ≤ 0.001). Hematologic indices were similarly compromised in the high-ALBI group, with significantly lower hemoglobin (8.6 vs. 9.8 g/dL) and platelet counts (129.2 vs. 183.8 × 10⁹/L), as well as higher RDW (16.7% vs. 15.6%) (all *P* < 0.001).

Clinical severity was significantly elevated in the high-ALBI group (median SOFA score 8.0 vs. 5.0; *P* < 0.001). Patients in the high-ALBI group were slightly younger (median 65.0 vs. 68.2 years; *P* = 0.027). Conversely, regarding chronic comorbidities, the high-ALBI group presented with a lower prevalence of heart failure (24.8% vs. 37.2%; *P* = 0.001), diabetes (31.0% vs. 38.8%; *P* = 0.045), and chronic kidney disease (20.2% vs. 28.0%; *P* = 0.026).

**Fig. 2:**
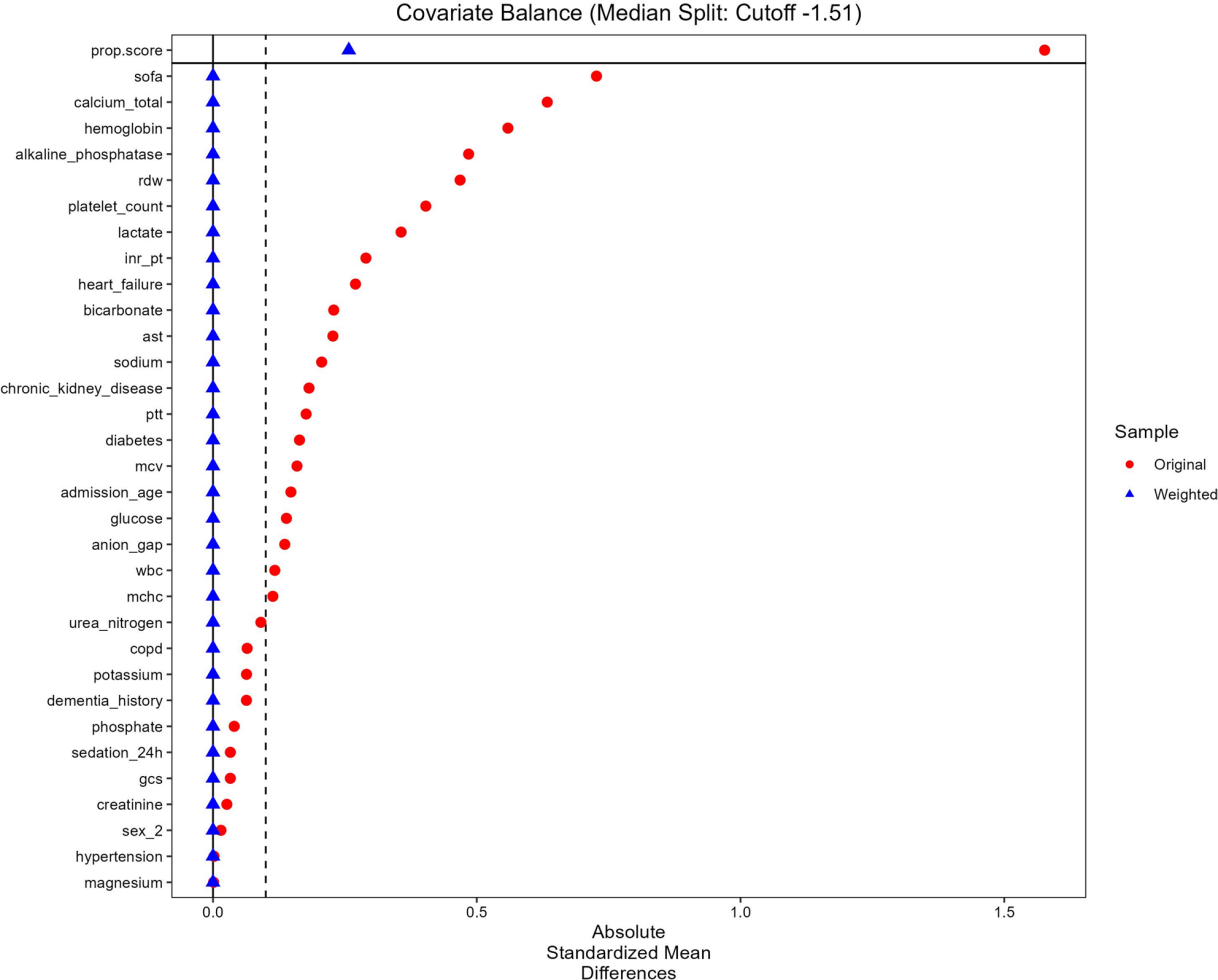
Covariate balance before and after CBPS-based inverse probability of treatment weighting by median albumin–bilirubin group. The x-axis displays the absolute standardized mean difference (SMD). Red circles represent the unadjusted cohort, and blue triangles represent the weighted cohort. The vertical dashed line indicates the threshold for balance (|SMD| = 0.10)

Covariate balancing via CBPS-based IPTW achieved excellent alignment across all baseline variables. Substantial baseline imbalances were effectively neutralized, with absolute SMDs reducing from high unweighted values (e.g., SOFA 0.727, calcium 0.634, Hb 0.559) to negligible levels after weighting (all IPTW SMDs < 0.001), strictly adhering to the prespecified balance criterion of |SMD| < 0.10 (Fig. 2; Table 1).

**Table 1:** Baseline characteristics of the study population stratified by albumin–bilirubin score

Variable	Overall (*n* = 651)	Low (*n* = 325)	High (*n* = 326)	*P*	Crude SMD	IPTW SMD
Gender, male	266 (40.9%)	134 (41.2%)	132 (40.5%)	0.911	0.015	< 0.001
Age, yr	66.2 [53.9–75.8]	68.2 [54.9–77.5]	65.0 [53.4–74.0]	0.027	0.148	< 0.001
Glucose, mg/dL	130.0 [108.4–162.8]	134.0 [110.0–163.8]	126.3 [106.0–161.6]	0.056	0.139	< 0.001
Hb, g/dL	9.2 [8.0–10.8]	9.8 [8.4–11.8]	8.6 [7.8–10.2]	< 0.001	0.559	< 0.001
Na, mmol/L	137.3 [134.0–140.5]	137.5 [134.7–140.8]	136.7 [133.7–140.0]	0.014	0.206	< 0.001
K, mmol/L	4.10 [3.83–4.52]	4.12 [3.83–4.54]	4.10 [3.83–4.50]	0.514	0.064	< 0.001
Cr, mg/dL	1.30 [0.87–2.22]	1.27 [0.85–2.15]	1.30 [0.90–2.29]	0.549	0.026	< 0.001
HCO₃⁻, mmol/L	22.0 [18.9–25.0]	22.8 [19.4–25.3]	21.2 [18.2–24.3]	0.001	0.229	< 0.001
BUN, mg/dL	29.0 [18.0–49.3]	26.3 [16.8–47.0]	31.5 [18.3–50.3]	0.05	0.091	< 0.001
AG, mmol/L	14.8 [12.5–17.7]	15.0 [12.8–17.8]	14.3 [12.0–17.2]	0.01	0.136	< 0.001
Lactate, mmol/L	2.05 [1.50–3.11]	1.90 [1.40–2.77]	2.29 [1.60–3.71]	< 0.001	0.357	< 0.001
Mg, mmol/L	2.02 [1.87–2.21]	2.05 [1.88–2.20]	2.00 [1.87–2.21]	0.572	0.001	< 0.001
PLT, 10⁹/L	157.3 [87.6–252.5]	183.8 [116.0–274.3]	129.2 [63.6–226.0]	< 0.001	0.403	< 0.001
Phos, mmol/L	3.60 [2.91–4.47]	3.55 [2.93–4.47]	3.63 [2.90–4.47]	0.709	0.04	< 0.001
Ca, mmol/L	8.27 [7.75–8.80]	8.53 [8.12–8.97]	8.02 [7.60–8.57]	< 0.001	0.634	< 0.001
WBC, 10⁹/L	13.2 [8.6–18.3]	12.7 [8.6–17.3]	13.6 [8.5–20.7]	0.061	0.117	< 0.001
RDW, %	16.2 [14.6–18.5]	15.6 [14.0–17.7]	16.7 [15.1–19.3]	< 0.001	0.468	< 0.001
MCHC, g/dL	32.5 [31.4–33.5]	32.4 [31.4–33.5]	32.5 [31.6–33.6]	0.216	0.113	< 0.001
MCV, fL	92.6 [88.0–97.6]	92.2 [87.5–97.0]	92.8 [88.8–98.5]	0.061	0.159	< 0.001
PTT, s	35.3 [29.3–49.2]	33.3 [28.5–44.9]	37.5 [31.1–53.4]	< 0.001	0.177	< 0.001
INR	1.50 [1.27–2.10]	1.40 [1.20–1.88]	1.60 [1.35–2.23]	< 0.001	0.29	< 0.001
AST, U/L	52.5 [28.0–106.8]	40.3 [24.3–78.3]	70.5 [34.5–138.6]	< 0.001	0.227	< 0.001
ALP, U/L	113.0 [75.0–185.3]	101.0 [69.5–144.0]	140.3 [91.8–240.1]	< 0.001	0.485	< 0.001
SOFA score	7.0 [4.0–10.0]	5.0 [3.0–8.0]	8.0 [5.0–12.0]	< 0.001	0.727	< 0.001
GCS score	15.0 [13.0–15.0]	15.0 [13.0–15.0]	15.0 [13.0–15.0]	0.679	0.033	< 0.001
DM	227 (34.9%)	126 (38.8%)	101 (31.0%)	0.045	0.164	< 0.001
HTN	186 (28.6%)	93 (28.6%)	93 (28.5%)	1	0.002	< 0.001
HF	202 (31.0%)	121 (37.2%)	81 (24.8%)	0.001	0.27	< 0.001
CKD	157 (24.1%)	91 (28.0%)	66 (20.2%)	0.026	0.182	< 0.001
COPD	60 (9.2%)	33 (10.2%)	27 (8.3%)	0.49	0.065	< 0.001
Dementia = 1 (%)	26 (4.0)	15 (4.6)	11 (3.4)	0.543	0.063	< 0.001
Analgo-sedation (%)	173 (26.6)	84 (25.8)	89 (27.3)	0.74	0.033	< 0.001
Clinical outcomes (%)				0.001	0.299	0.299
Censored	281 (43.2)	164 (50.5)	117 (35.9)			
Death	42 (6.5)	17 (5.2)	25 (7.7)			
Delirium	328 (50.4)	144 (44.3)	184 (56.4)			

Baseline characteristics of the study population stratified by albumin–bilirubin score. Values are presented as median [interquartile range] or number (percentage). P values compare differences between groups in the unadjusted cohort. SMD < 0.1 indicates negligible imbalance between groups. Abbreviations: AG, anion gap; ALBI, albumin-bilirubin; ALP, alkaline phosphatase; AST, aspartate aminotransferase; BUN, blood urea nitrogen; Ca, total calcium; CKD, chronic kidney disease; COPD, chronic obstructive pulmonary disease; Cr, creatinine; DM, diabetes mellitus; GCS, Glasgow Coma Scale; Hb, hemoglobin; HCO₃⁻, bicarbonate; HF, heart failure; HTN, hypertension; INR, international normalized ratio; IPTW, inverse probability of treatment weighting; K, potassium; MCHC, mean corpuscular hemoglobin concentration; MCV, mean corpuscular volume; Mg, magnesium; Na, sodium; Phos, phosphate; PLT, platelet count; PTT, partial thromboplastin time; RDW, red cell distribution width; SMD, standardized mean difference; SOFA, Sequential Organ Failure Assessment; WBC, white blood cell. Analgo-sedation was defined as the administration of midazolam, lorazepam, propofol, fentanyl, or morphine within 24 h prior to assessment

### Covariate balance

The assessment of covariate balance before and after weighting is visualized in the Love plot (Fig. 2). In the original unadjusted cohort, substantial imbalances existed between the low- and high-ALBI groups. As indicated by the red circles, the absolute SMDs for multiple covariates exceeded the threshold of 0.10, with the most pronounced disparities observed in the propensity score, SOFA score, Ca, Hb, and ALP. Following the application of CBPS-based IPTW, these imbalances were effectively mitigated. As shown by the blue triangles in Fig. 2, the SMDs for all covariates converged closely to zero and were well below the 0.10 threshold, confirming that excellent baseline balance was achieved between the two groups.

### Cumulative incidence of delirium

**Fig. 3:**
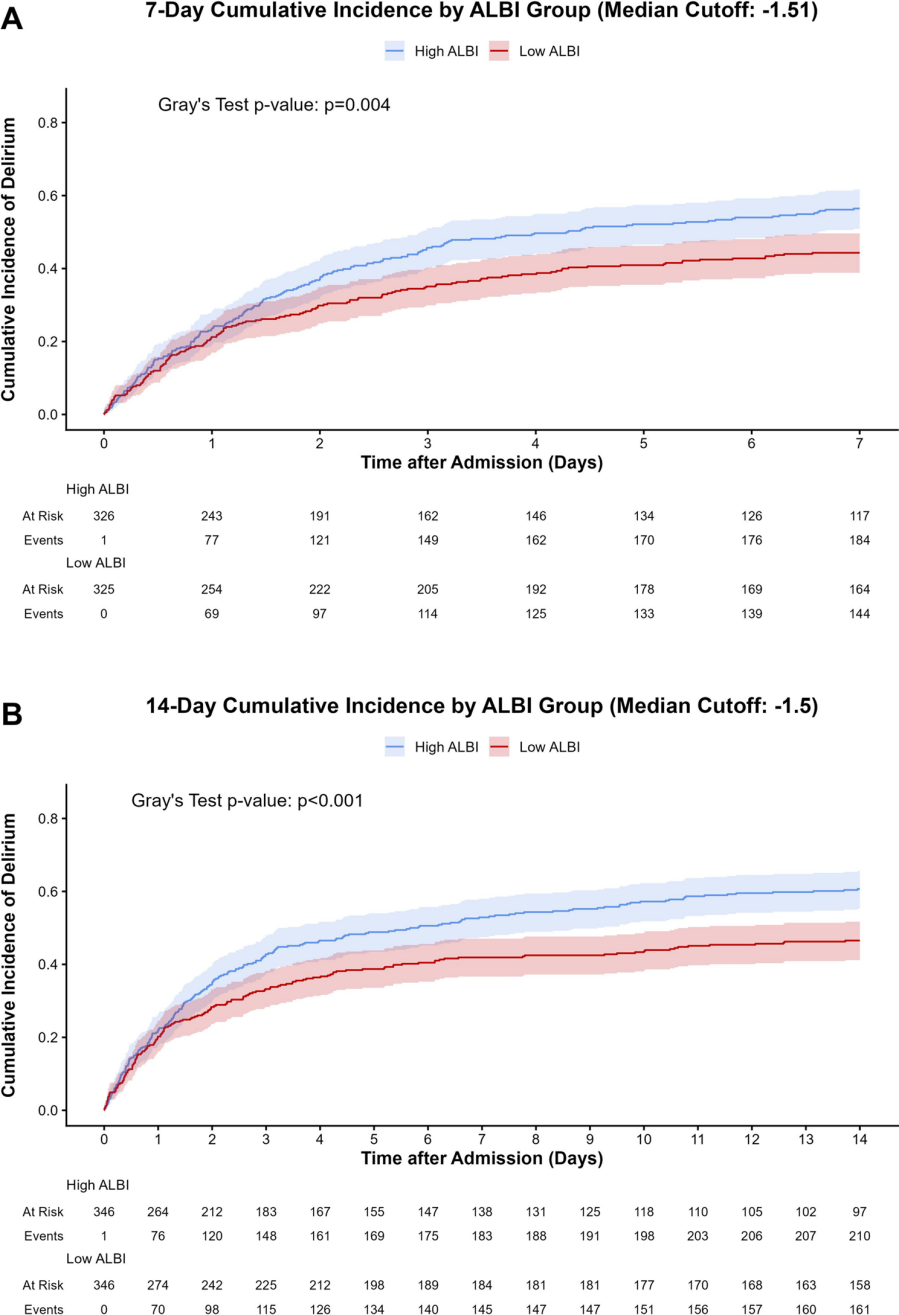
Cumulative incidence of delirium stratified by the median albumin-bilirubin score following bloodstream infection. Cumulative incidence functions (CIF) for the onset of delirium are shown for the high-ALBI (blue line) and low-ALBI (red line) groups, accounting for death as a competing risk. Shaded areas represent 95% confidence intervals. (**A**) CIF at 7 days post-bloodstream infection (Gray’s test *P* = 0.004). (**B**) CIF at 14 days post-bloodstream infection (Gray’s test *P* < 0.001). The risk tables below the graphs indicate the number of patients at risk and the cumulative number of delirium events at each time point

The cumulative incidence of delirium was analyzed using a competing risks framework, treating death as a competing event. Figure 3 illustrates the cumulative incidence functions (CIF) stratified by the median ALBI score. Patients in the high-ALBI group exhibited a significantly elevated risk of developing delirium compared to those in the low-ALBI group. At 7 days, the cumulative incidence was markedly higher in the high-ALBI group (56.4% vs. 44.3%; Gray’s test *P* = 0.004; Fig. 3A). This disparity persisted and the curves showed continued divergence over the observation period; by 14 days, the difference in delirium incidence between the high- and low-ALBI groups remained highly significant (60.7% vs. 46.5%; Gray’s test *P* < 0.001; Fig. 3B). Visual inspection indicates an early separation of the curves, suggesting that elevated ALBI scores are associated with an increased risk of delirium onset shortly after the diagnosis of bloodstream infection.

### Variable selection for multivariable analysis

**Fig. 4:**
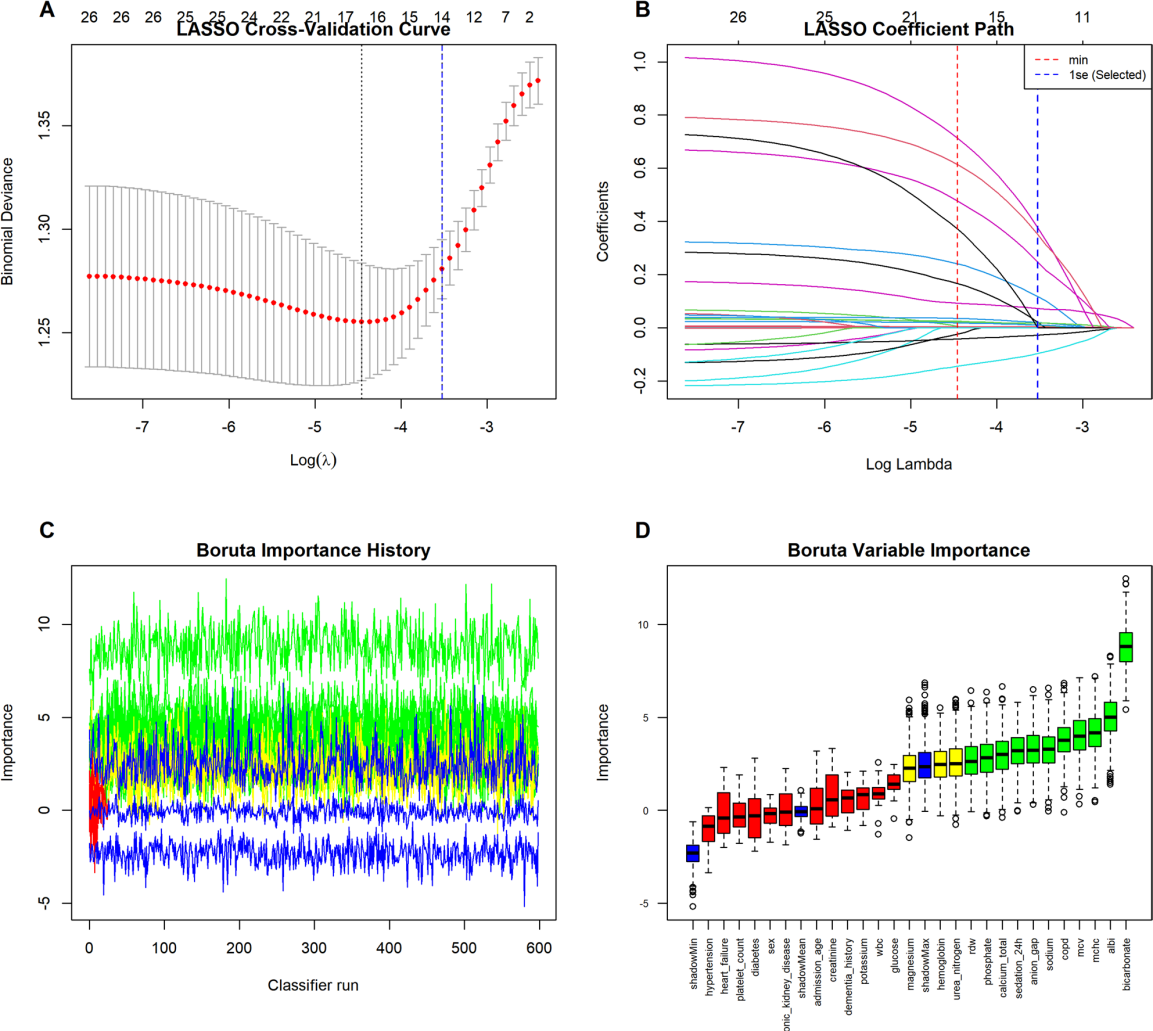
Feature selection process for the multivariable model using a dual-algorithm approach. (**A**) LASSO cross-validation curve; the vertical blue dashed line indicates the penalty parameter (λ) within 1 standard error of the minimum (λ.1se). (**B**) LASSO coefficient profiles plotted against log(λ). (**C**) Boruta attribute importance history over random forest iterations. (**D**) Boruta variable importance plot; green and blue boxplots represent confirmed features and shadow features (significance thresholds), respectively. The final model covariates were determined by the intersection of LASSO and Boruta selections, supplemented by clinically pre-specified variables

To construct a robust multivariable Fine-Gray model, we employed a hybrid covariate selection strategy that integrated data-driven algorithms with clinical expertise. As illustrated in Fig. 4, the initial screening utilized both LASSO regression and the Boruta algorithm. The LASSO analysis, applying the stricter lambda.1se criterion, effectively shrank less informative coefficients to zero (Fig. 4A and B). Concurrently, the Boruta algorithm evaluated variable importance relative to shadow features (Fig. 4C and D).

The intersection of these two algorithms identified 12 key data-driven predictors, including the ALBI score, analgo-sedation, COPD, and specific laboratory indices (MCHC, MCV, RDW, total calcium, phosphate, anion gap, blood urea nitrogen, bicarbonate, and sodium). To ensure clinical interpretability and adequately control for established confounders, we additionally included age, gender, history of dementia, and other major comorbidities (diabetes mellitus, hypertension, heart failure, and chronic kidney disease) into the final model a priori, supplementing the algorithmic selection. Consequently, the final multivariable Fine-Gray model was adjusted for this comprehensive set of demographic, clinical, and laboratory covariates.

### Dose-response relationship

To further characterize the dose-response relationship between the continuous ALBI score and the risk of delirium, RCS analyses were performed based on the Fine-Gray proportional subdistribution hazard models. The models were fully adjusted for age, gender, major comorbidities (diabetes, hypertension, heart failure, CKD, COPD, and history of dementia), analgo-sedation, and specific laboratory parameters (MCHC, MCV, RDW, total calcium, phosphate, anion gap, BUN, bicarbonate, and sodium).

**Fig. 5:**
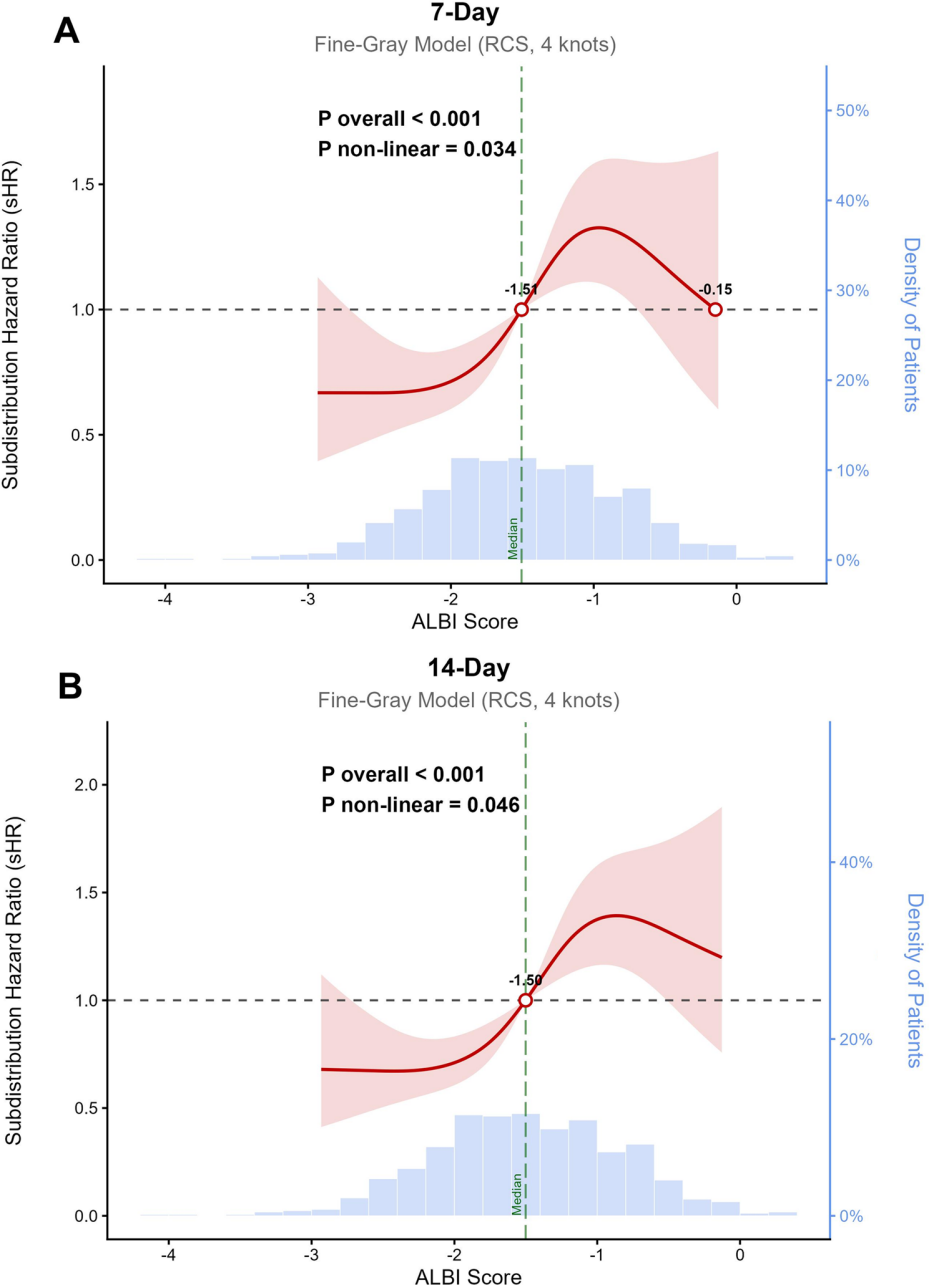
Restricted cubic spline analysis of the association between albumin–bilirubin score and the risk of incident delirium. The association was modeled using Fine-Gray proportional subdistribution hazard models with 4 knots. Models were adjusted for age, gender, analgo-sedation, comorbidities (diabetes, hypertension, heart failure, dementia history, CKD, COPD), and laboratory indices (MCHC, MCV, RDW, total calcium, phosphate, anion gap, BUN, bicarbonate, sodium). The median ALBI score served as the reference (sHR = 1.00, white circle). The solid red line represents the sHR, and the pink area indicates the 95% CI. Blue histograms show the ALBI score distribution. (**A**) 7-day incident delirium (P for overall association < 0.001, P for non-linearity = 0.034). (**B**) 14-day incident delirium (P for overall association < 0.001, P for non-linearity = 0.046). Abbreviations: ALBI, albumin-bilirubin; BUN, blood urea nitrogen; CKD, chronic kidney disease; COPD, chronic obstructive pulmonary disease; MCHC, mean corpuscular hemoglobin concentration; MCV, mean corpuscular volume; RDW, red cell distribution width; sHR, subdistribution hazard ratio

For the 7-day outcome (Fig. 5A), the RCS analysis revealed a significant non-linear association between the ALBI score and the risk of incident delirium (P for overall association < 0.001; P for non-linearity = 0.034). Using the median ALBI score (-1.51) as the reference, the risk of delirium progressively increased with rising ALBI scores. A J-shaped trend was observed, where the sHR escalated sharply as the ALBI score exceeded the reference value.

For the 14-day outcome (Fig. 5B), results were consistent. A significant overall association was observed (P for overall association < 0.001), and the relationship exhibited significant non-linearity (P for non-linearity = 0.046). Similar to the 7-day model, the sHR for delirium increased continuously with ALBI scores above the median reference value (-1.50).

### Association between ALBI score and delirium risk (fine-gray regression)

The association between the ALBI score and the risk of incident delirium at 7 and 14 days was evaluated using IPTW-weighted Fine-Gray proportional subdistribution hazard models. The results are presented in Table 2.

7-Day Delirium In the unadjusted model (Model 1), a one-unit increase in the continuous ALBI score was significantly associated with an elevated risk of 7-day delirium (sHR 1.46, 95% CI 1.17–1.83, *P* < 0.001). This association remained consistent after adjusting for age and gender in Model 2 (sHR 1.45, 95% CI 1.15–1.83, *P* = 0.002). Crucially, in the fully adjusted model (Model 3), which controlled for gender, multiple comorbidities, analgo-sedation, and several laboratory parameters, the ALBI score remained an independent and stronger predictor of 7-day delirium, showing a substantial risk increase per one-unit rise (sHR 1.80, 95% CI 1.38–2.33, *P* < 0.001). Furthermore, when the ALBI score was analyzed as a categorical variable, the high-ALBI group (compared to the low-ALBI group) showed a significantly higher subdistribution hazard ratio (sHR 1.98, 95% CI 1.38–2.83, *P* < 0.001) in Model 3. The P for trend was highly significant across all models, confirming a dose-response relationship (*P* < 0.001 for Model 3).

14-Day Delirium Similar trends were observed for the 14-day delirium outcome. In the unadjusted and age- and gender-adjusted models (Model 1 and Model 2), the continuous ALBI score was significantly associated with increased risk (sHR 1.44, 95% CI 1.13–1.83, *P* = 0.003 and sHR 1.45, 95% CI 1.13–1.86, *P* = 0.003, respectively). In the fully adjusted model (Model 3), the ALBI score maintained its robust, independent association with 14-day delirium (sHR 1.66, 95% CI 1.26–2.19, *P* < 0.001). Categorical analysis also showed that the high-ALBI group had a significantly elevated risk of 14-day delirium (sHR 1.87, 95% CI 1.30–2.68, *P* < 0.001) compared to the low-ALBI group, with a significant P for trend across all models (*P* < 0.001 for Model 3).

**Table 2:** IPTW-weighted fine-gray regression analyses of the association between ALBI Score and delirium at 7 and 14 days

Variables	Model 1 sHR (95% CI)	*P*-value	Model 2 sHR (95% CI)	*P*-value	Model 3 sHR (95% CI)	*P*-value
7-day delirium						
Per 1-unit increase	1.46 (1.17–1.83)	< 0.001	1.45 (1.15–1.83)	0.002	1.80 (1.38–2.33)	< 0.001
Low	1 (Reference)	–	1 (Reference)	–	1 (Reference)	–
High	1.73 (1.23–2.43)	0.002	1.72 (1.23–2.43)	0.002	1.98 (1.38–2.83)	< 0.001
P for trend		0.002		0.002		< 0.001
14-day delirium						
Per 1-unit increase	1.44 (1.13–1.83)	0.003	1.45 (1.13–1.86)	0.003	1.66 (1.26–2.19)	< 0.001
Low	1 (Reference)	–	1 (Reference)	–	1 (Reference)	–
High	1.69 (1.17–2.43)	0.005	1.69 (1.17–2.43)	0.005	1.87 (1.30–2.68)	< 0.001
P for trend		0.005		0.005		< 0.001

Model 1: Unadjusted

Model 2: Adjusted for age and gender

Model 3: Adjusted for age, gender, analgo-sedation, comorbidities (diabetes mellitus, hypertension, heart failure, history of dementia, chronic kidney disease, COPD), and laboratory parameters (MCHC, MCV, RDW, total calcium, phosphate, anion gap, blood urea nitrogen, bicarbonate, and sodium)

Abbreviations: ALBI, albumin-bilirubin; CI, confidence interval; COPD, chronic obstructive pulmonary disease; MCHC, mean corpuscular hemoglobin concentration; MCV, mean corpuscular volume; RDW, red cell distribution width; sHR, subdistribution hazard ratio

### Subgroup analysis

**Fig. 6:**
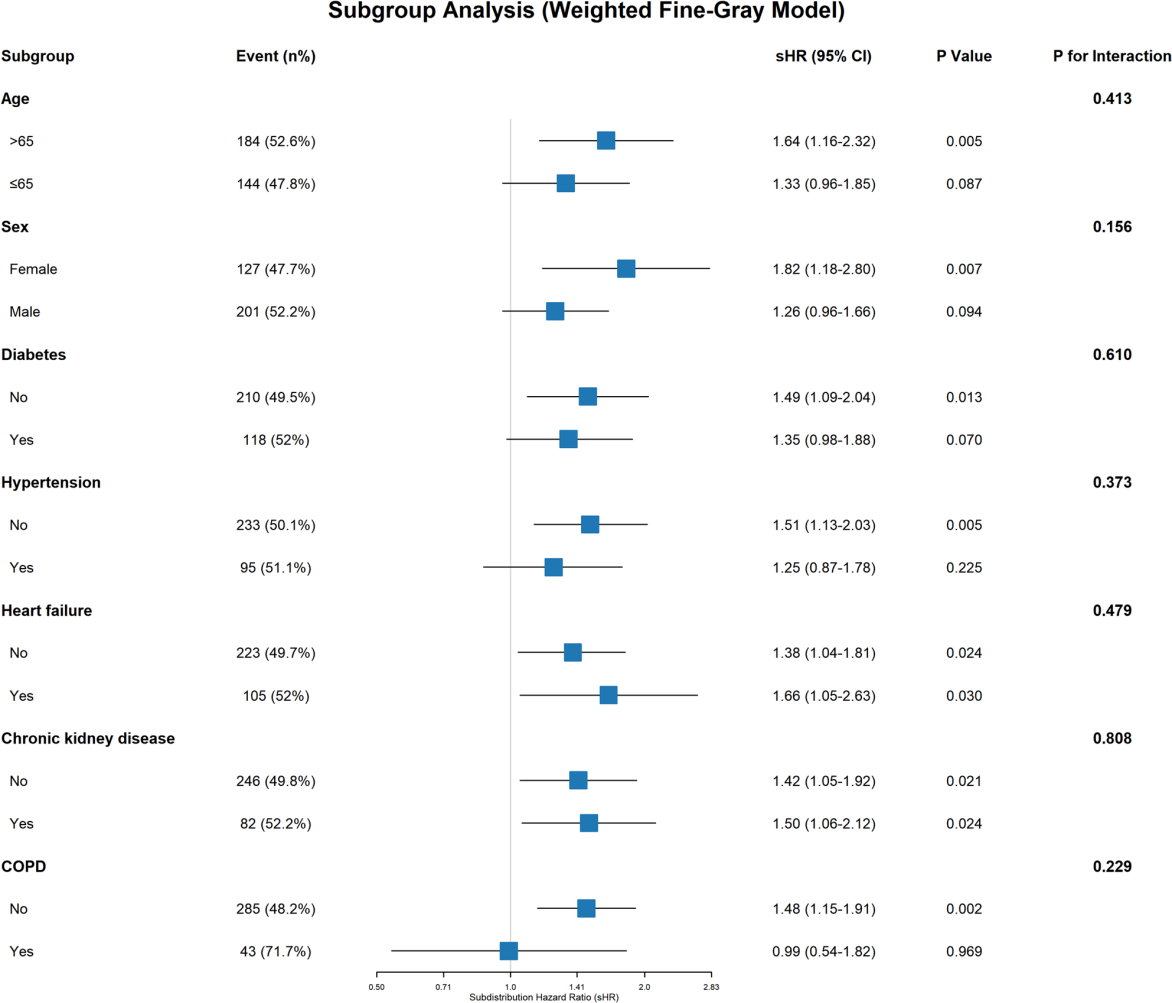
Subgroup analysis of the association between continuous albumin-bilirubin score and the risk of delirium using weighted Fine-Gray models. The analysis was stratified by age, gender, and comorbidities (heart failure, chronic kidney disease, and COPD). The squares represent the point estimates of the subdistribution hazard ratio (sHR), and the horizontal lines indicate the 95% confidence interval (CI). P values for interaction evaluate the heterogeneity of the ALBI score association across subgroups. Abbreviations: ALBI, albumin-bilirubin; CI, confidence interval; COPD, chronic obstructive pulmonary disease; sHR, subdistribution hazard ratio

To test the robustness of the association between the ALBI score and the risk of delirium, weighted Fine-Gray regression was performed across various subgroups (Fig. 6). The association between the ALBI score and the risk of delirium was largely consistent across all stratified analyses, with no significant effect modification observed (all P for interaction > 0.05).

Regarding age, the association appeared numerically stronger in patients aged ≤ 65 years (sHR 1.64, 95% CI 1.16–2.32, *P* = 0.005) compared to those aged > 65 years (sHR 1.33, 95% CI 0.96–1.85, *P* = 0.087); however, the interaction was not statistically significant (P for interaction = 0.413).

Regarding gender, the association was more pronounced in females (sHR 1.82, 95% CI 1.18–2.80, *P* = 0.007) than in males (sHR 1.26, 95% CI 0.96–1.66, *P* = 0.094), though the difference between groups was not statistically significant (P for interaction = 0.156).

In terms of comorbidities, the prognostic value of the ALBI score remained significant regardless of chronic kidney disease status (No: *P* = 0.021; Yes: *P* = 0.024) or heart failure status (No: *P* = 0.024; Yes: *P* = 0.030). Although the association was not statistically significant in patients with COPD (sHR 0.99, *P* = 0.969), likely due to the limited sample size in this subgroup, the overall interaction test did not indicate a significant difference (P for interaction = 0.229).

## Discussion

In this retrospective cohort study of patients with BSI, we utilized a propensity-weighted competing-risks analysis to evaluate the association between the ALBI score and the risk of incident delirium. Our findings indicate that an elevated ALBI score is independently associated with a significant increase in the risk of developing delirium within 7 days of BSI diagnosis. This association persisted even after rigorous adjustment for demographic characteristics, major comorbidities, and analgo-sedation exposure using Fine-Gray proportional subdistribution hazard models. Specifically, a non-linear, J-shaped dose-response relationship was identified, where increasing ALBI scores correlated with a progressively higher subdistribution hazard ratio. These results suggest that the ALBI score, a routinely available assessment of hepatic function, may serve as a potential objective indicator for stratifying the risk of delirium following bloodstream infection.

Delirium remains common after bloodstream infection, with an incidence higher than that observed in general ICU populations [18], yet validated early biomarkers are relatively scarce. Previous reports have linked hypoalbuminemia to an increased risk of delirium in critically ill adults [19] and to worse sepsis outcomes [20], supporting albumin as a prognostic indicator. However, other studies have reported no significant association between albumin levels and either the occurrence or severity of delirium [21]. In contrast, the ALBI score—combining albumin and bilirubin—has demonstrated prognostic value for mortality in ICU sepsis populations [22], and our findings indicate that the ALBI score may hold potential utility for identifying delirium risk within the first week after BSI. Biologically, bilirubin-related neurotoxicity and BBB vulnerability observed in hepatic dysfunction further support the plausibility that a composite reflecting both hypoalbuminemia and hyperbilirubinemia could capture delirium susceptibility [23, 24].

ALBI integrates hypoalbuminemia and hyperbilirubinemia—two signals plausibly linked to acute brain dysfunction in sepsis. Albumin has non-oncotic, neurovascularly relevant functions, including anti-inflammatory and antioxidant activity [25, 26], ligand binding/detoxification [27], and endothelial–glycocalyx stabilization [28]; thus, lower albumin may diminish antioxidant buffering capacity, exacerbate endothelial leakage, and indirectly promote BBB vulnerability. In addition, extravasated albumin may induce the expression of proinflammatory cytokines and impair astrocytic function, which in turn further contributes to reduced circulating albumin levels [29]. Although bilirubin can exert antioxidant effects at physiologic levels [30], elevated unconjugated bilirubin is able to cross the BBB and exert neurotoxic effects on both glia and neurons [31]. Clinically, higher bilirubin also reflects underlying hepatic dysfunction and systemic inflammatory stress [32], and worse hepatic function may predispose patients to delirium [33, 34]. Mechanistically, this association may involve the ‘liver–brain axis.’ The liver serves as a critical organ for the clearance of circulating neurotoxins (e.g., ammonia, aromatic amino acids) and inflammatory mediators [35]. When hepatic function is compromised—as signaled by an elevated ALBI score—this clearance capacity is impaired, leading to the accumulation of neurotoxic substances [36]. Crucially, sepsis-induced systemic inflammation compromises the blood-brain barrier, allowing these accumulated neurotoxins to penetrate the central nervous system (CNS) [37, 38] and act synergistically with sepsis-induced systemic inflammation to activate cerebral microglia via neurohumoral pathways, exacerbating neuroinflammation and neurotransmitter dysregulation [39]. Ultimately, this cascade may induce a pathological state analogous to ‘subclinical hepatic encephalopathy,’ thereby increasing susceptibility to delirium. Taken together, a higher ALBI (combining lower albumin with higher bilirubin) may capture compounded oxidative–inflammatory stress and impaired endothelial/BBB integrity, offering a biologically coherent explanation for the observed increase in 7-day delirium risk.

The positive association between the ALBI score and delirium risk was generally consistent across prespecified subgroups, including those stratified by age, gender, and major comorbidities. Furthermore, interaction analyses revealed no significant effect modification by specific chronic conditions, such as COPD (P for interaction = 0.229). This lack of significant interaction suggests that the ALBI score maintains its prognostic value across a heterogeneous population of patients with bloodstream infections, largely independent of their baseline comorbidity profile.

ALBI is derived from two universally available tests—albumin and bilirubin—and was originally developed as an objective, granular index of hepatic function [8]; its use has since expanded beyond hepatology [40]. Emerging ICU data indicate that higher ALBI portends worse outcomes in sepsis populations [41], aligning with our delirium-focused findings. Because delirium is associated with increased mortality, prolonged length of stay, and other adverse outcomes, early and objective identification of high-risk patients is of clear clinical importance [42]. While we demonstrated a strong independent association using robust causal inference methods (IPTW), we did not formally evaluate the predictive performance metrics (e.g., area under the curve, calibration) or develop a prediction model. Our primary objective was to elucidate the independent etiological association between hepatic dysfunction (via ALBI) and delirium using a competing-risks framework, rather than to derive and validate a de novo clinical prediction score. Therefore, the ALBI score should currently be viewed as a valuable complementary signal to flag high-risk patients rather than a standalone diagnostic tool. In practice, given its automated nature, ALBI could complement existing bedside risk assessments to prioritize limited resources and trigger preventive bundles (e.g., analgesia/sedation optimization, early mobilization, family engagement) consistent with PADIS (Pain, Agitation/Sedation, Delirium, Immobility, and Sleep disruption) and ICU Liberation approaches, providing a pragmatic strategy to improve care although the specific effects on delirium have varied across studies [43].

This study has several limitations inherent to its retrospective design. First, observational studies cannot establish causality. To address this, we employed rigorous CBPS-based IPTW to maximize covariate balance, in an effort to minimize the influence of measured confounders. Second, detailed dosing of sedatives and baseline cognitive scores were not available. Nevertheless, our models adjusted for the presence of analgo-sedation and history of dementia to help mitigate potential bias. Third, a proportion of patients were excluded due to missing laboratory values. However, our comparison of baseline characteristics (Supplementary Table S1) suggests that the included cohort remains largely representative of the target population. Fourth, delirium was ascertained using the validated CAM-ICU tool. While continuous monitoring is ideal, CAM-ICU is the standard of care in the MIMIC-IV database and is generally accepted for large-scale epidemiological research. Finally, given the multifactorial etiology of delirium, the ALBI score may be best utilized as a component of multimodal risk stratification rather than a standalone predictive tool. Future research should focus on integrating ALBI into comprehensive prediction models, evaluating dynamic ALBI trajectories to capture disease progression, and conducting external validation in multicenter cohorts and diverse clinical settings (including lower-resource ICUs) to confirm generalizability before widespread clinical application.

## Conclusion

Our findings suggest that in critically ill adults with BSI, an elevated baseline ALBI score is independently associated with an increased risk of 7-day incident delirium, characterized by a non-linear, J-shaped relationship. Derived from routinely available liver function tests, the ALBI score shows promise as a potential objective marker to flag high-risk patients in the ICU, complementing existing clinical assessments. For patients with elevated ALBI, it may be prudent to consider the early implementation of evidence-based delirium-prevention bundles (e.g., analgesia/sedation optimization, early mobilization, reorientation, family engagement) in an effort to mitigate risk and improve outcomes.

## Supplementary Information

Below is the link to the electronic supplementary material.

Supplementary Material 1

Supplementary Material 2

Supplementary Material 3

## Data Availability

This study used the publicly available Medical Information Mart for Intensive Care IV (MIMIC-IV) database (version 3.1), hosted on PhysioNet (10.13026/kpb9-mt58). Access to the de-identified data requires completion of human-subjects protection training and signing of the PhysioNet Credentialed Data Use Agreement. The dataset can be accessed at https://physionet.org/content/mimiciv/.
