# Supplementary figures and images for: Association of the Albumin–Bilirubin score with 7-day incident delirium risk following bloodstream infection in critically ill adults: evidence from a propensity-weighted cohort
Source: BMC Infect Dis. 2026 Apr 3;26:954. doi: 10.1186/s12879-026-13228-3 (PMC13173885; doi:10.1186/s12879-026-13228-3)

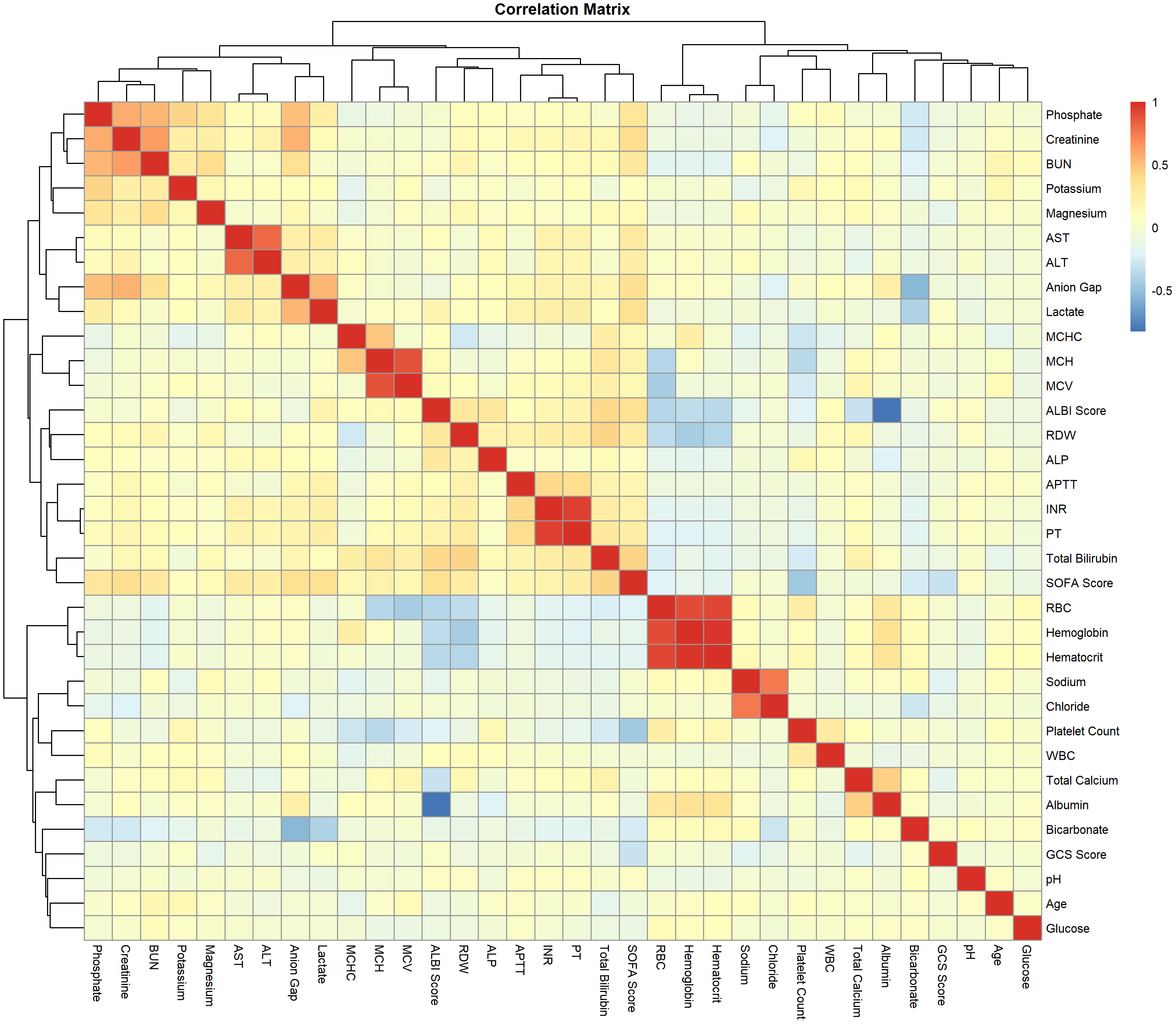

Supplement: Supplementary file 1 — Supplementary Material 1 [file 12879_2026_13228_MOESM1_ESM.tif]
