# Supplementary material for: Association of the Albumin–Bilirubin score with 7-day incident delirium risk following bloodstream infection in critically ill adults: evidence from a propensity-weighted cohort
Source: BMC Infect Dis. 2026 Apr 3;26:954. doi: 10.1186/s12879-026-13228-3 (PMC13173885; doi:10.1186/s12879-026-13228-3)
Supplement: Supplementary file 2 — Supplementary Material 2 [file 12879_2026_13228_MOESM2_ESM.docx]

| Table S1. Comparison of baseline characteristics between included patients and those excluded due to missing data | | | |
| --- | --- | --- | --- |
| Variable | Included Cohort  (n = 651) | Excluded (Missing Data)  (n = 532) | P-value |
| Age, median [IQR] | 66.2 [53.9–75.8] | 68.7 [59.1–77.4] | 0.002 |
| Male sex, n (%) | 266 (40.9%) | 208 (39.1%) | 0.578 |
| SOFA score, median [IQR] | 7.0 [4.0–10.0] | 5.0 [3.0–8.0] | <0.001 |
| GCS score, median [IQR] | 15.0 [13.0–15.0] | 15.0 [13.0–15.0] | 0.296 |
| Comorbidities, n (%) |  |  |  |
| Diabetes | 227 (34.9%) | 186 (35.0%) | 1 |
| Hypertension | 186 (28.6%) | 176 (33.1%) | 0.107 |
| Heart Failure | 202 (31.0%) | 190 (35.7%) | 0.101 |
| Chronic Kidney Disease | 157 (24.1%) | 145 (27.3%) | 0.244 |
| COPD | 60 (9.2%) | 62 (11.7%) | 0.202 |
| Primary Outcome |  |  |  |
| Delirium (fstatus=2), n (%) | 328 (50.4%) | 249 (46.8%) | 0.27 |
| Data are presented as median [interquartile range (IQR)] for continuous variables and frequency (percentage) for categorical variables. P-values indicate differences between the included and excluded groups. Abbreviations: SOFA, Sequential Organ Failure Assessment; GCS, Glasgow Coma Scale; COPD, Chronic Obstructive Pulmonary Disease; IQR, interquartile range. | | | |
